# Rapidly growing Fibro-osseous pseudotumor of the digit: A case report

**DOI:** 10.1097/MD.0000000000021116

**Published:** 2020-07-10

**Authors:** Tomohiko Sakuda, Tadahiko Kubo, Rikuo Shinomiya, Taisuke Furuta, Nobuo Adachi

**Affiliations:** Department of Orthopaedic Surgery, Graduate School of Biomedical and Health Sciences, Hiroshima University, Minami-ku, Hiroshima, Japan.

**Keywords:** benign lesion, fasciitis ossificans, fibro-osseous pseudotumor of the digit, pseudomalignancy, subcutaneous tissue

## Abstract

**Introduction:**

Fibro-osseous pseudotumor of the digit is a rare benign lesion of subcutaneous tissue that typically arises in the parabone site of the proximal phalanx in young adult females. The lesion is histopathologically characterized by fibroblastic proliferation and osteoid formation. Good prognosis following complete surgical excision of the tumor has been reported, with a very low recurrence rate and no reports of malignant transformation. Despite its benign clinical behavior, the lesion can be mistaken for a malignant neoplasm, such as an extraskeletal or parosteal osteosarcoma, in case of rapid growth, thereby rendering the diagnosis challenging.

**Patient concerns:**

We report the case of a 30-year-old right-handed male who presented to our hospital with a rapidly growing mass on the dorsal aspect of the right little finger.

**Diagnosis:**

The patient was suspected to have soft tissue tumor of the little finger. The lesion could be considered a malignant tumor on the basis of clinical findings.

**Interventions:**

The patient underwent surgery for exploration and excision of the mass.

**Outcomes:**

The excised mass was diagnosed to be fibro-osseous pseudotumor of the digit upon histological assessment. Postoperatively, the wound healed without complications. At postoperative 6 months, there were no signs or symptoms of recurrence, and the patient returned to his premorbid functional status.

**Conclusion:**

Following the detection of a soft tissue mass with clinicopathological features of pseudomalignancy in the digit, clinicians should consider fibro-osseous pseudotumor of the digit as a possible diagnosis, thereby avoiding unnecessary aggressive surgery.

## 1 Introduction

Fibro-osseous pseudotumor of the digit (FOPD) is a rare benign lesion of subcutaneous tissue that typically arises in the parabone site of the proximal phalanx in young adult females. The lesion is characterized by fibroblastic proliferation and osteoid formation.^[1,2]^ FOPD usually presents as a localized proximal nodule and predominantly affects females. Following complete surgical excision, the lesion is associated with good prognosis, with a very low recurrence rate and no reports of malignant transformation.^[1,3]^ Despite it being benign, this lesion may be mistaken for a malignant neoplasm, such as an extraskeletal or parosteal osteosarcoma, in case of rapid growth. Here we report a rare and unusual case of a rapidly growing FOPD. The lesion could be considered a malignant tumor on the basis of clinical findings.

## 2 Case report

**Figure 1:**
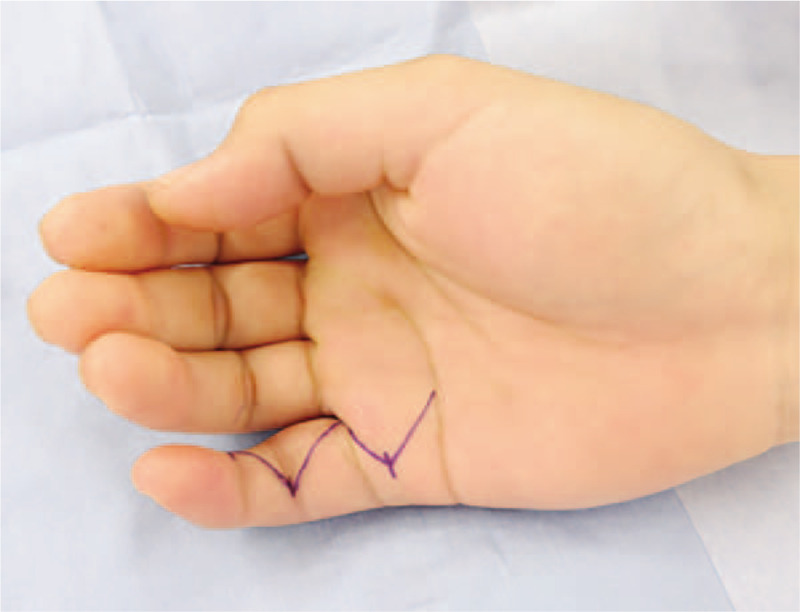
Clinical presentation of fibro-osseous pseudotumor of the digit. It shows the preoperative clinical presentation of the mass at the base of the right little finger. Preoperative incision marking is also shown.

A 30-year-old right-handed male presented to our hospital with a painless palpable growing mass on the dorsal aspect of the right little finger. He had noticed the mass 6 weeks prior to consultation with us and had previously visited another hospital. Initially, a soft tissue tumor of the little finger was suspected, and a resection biopsy was performed for confirmation 4 weeks prior to our consultation. The biopsy revealed FOPD. Following rapid growth of the mass lesion, the patient was referred to our clinic. There was severe swelling on the base of the little finger; however, there was no redness, local heat, or constitutional symptoms (such as fever, weight loss, or malaise) (Fig. 1). The patient did not recall any specific history of trauma; however, occupational injury due to an excessive load on the little finger was suspected.

**Figure 2:**
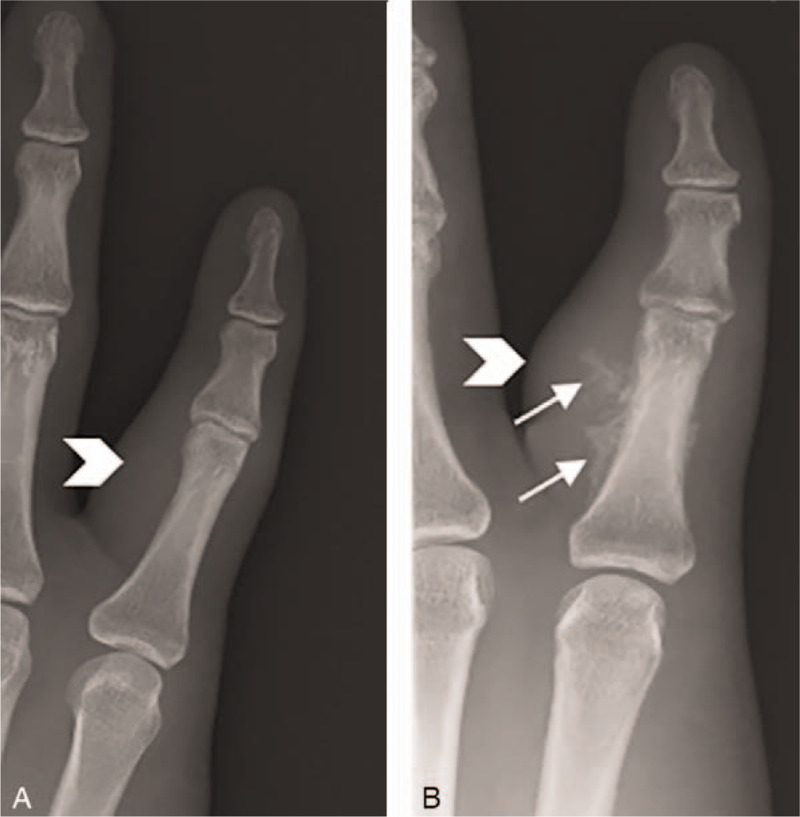
Radiological investigation during the first visit to another hospital (A) and our hospital (B). (A) Mild swelling (arrowhead) around the proximal phalanx of the little finger without calcification. (B) Severe swelling (arrowhead) around the proximal phalanx of the little finger with some calcification (arrow).

**Figure 3:**
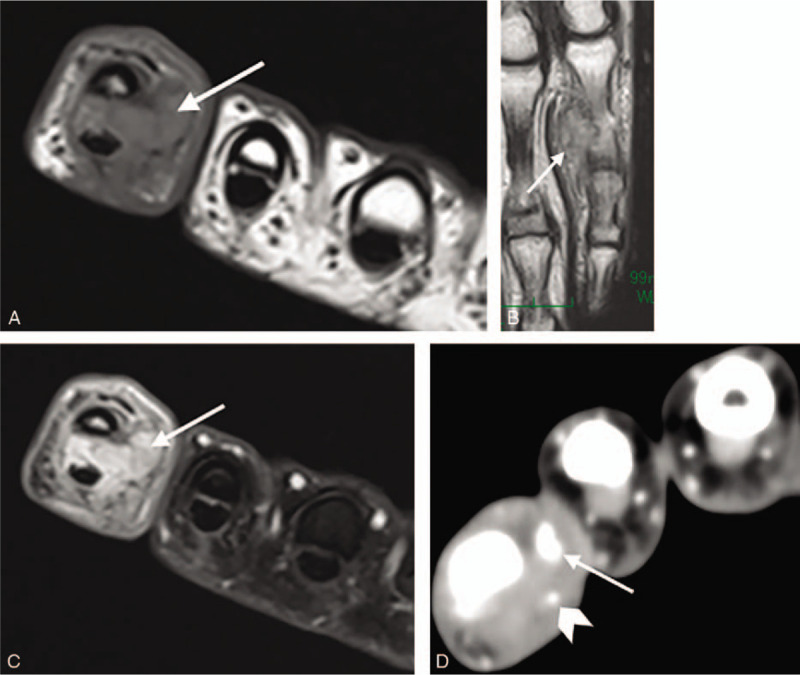
MRI T1-weighted axial view (A) demonstrating that the signal intensities of the lesion were homogenous hypointense (arrow). T2-weighted coronal view (B) demonstrating that the signal intensities of the lesion were heterogenous hyperintense (arrow). Contrast-enhanced T1-weighted axial view (C) demonstrating an enhanced T-shaped mass (arrow). Contrast-enhanced CT axial image (D) demonstrating that the mass was adjacent to the radial neurovascular bundle (arrowhead), and the surrounding calcifications were not continuous with the proximal phalanx bone (arrow). CT = computed tomography, MRI = magnetic resonance imaging.

A plain radiograph taken at the previous hospital revealed a mild soft tissue swelling without calcification or periosteal reaction (Fig. 2A). At the time of patient's first visit to us, a plain radiograph of the involved hand was obtained, revealing severe soft tissue swelling and some calcifications without evidence of bone involvement (Fig. 2B). Further evaluation through magnetic resonance imaging revealed a mass on the volar aspect of that digit, with low signal intensity on T1 and heterogenous high signal intensity on T2 evaluations (Fig. 3A and B). After the administration of gadolinium contrast agent, the lesions exhibited contrast enhancement on the T1-weighted image (Fig. 3C). On the contrast-enhanced T1-weighted axial view, the lesion was recognized as a T-shaped mass. It measured 1.5 × 2.5 × 1 cm, and had no connection with the underlying bone. Using contrast-enhanced computed tomography, the assessment of neurovascular structures showed partial abutment of the radial-sided bundle. In addition, the surrounding calcifications were not continuous with the proximal phalanx bone (Fig. 3D).

**Figure 4:**
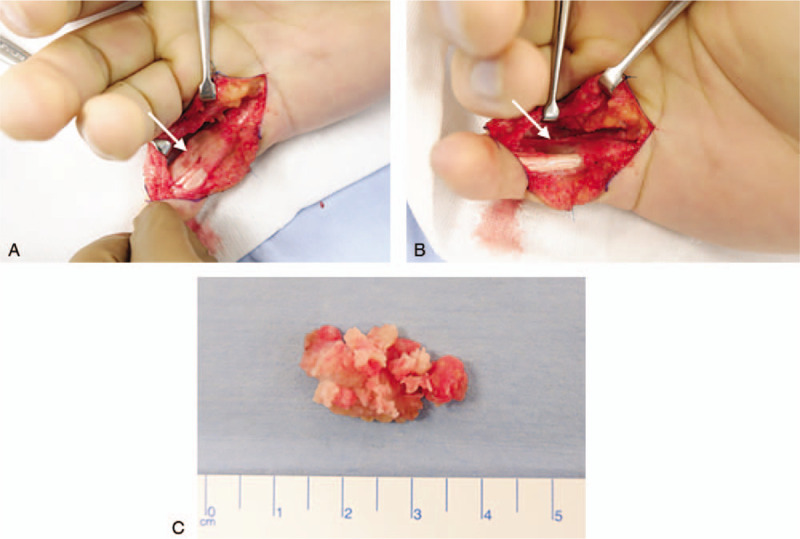
Intra-operative fibro-osseous pseudotumor excision. (A) Halved fibro-osseous pseudotumor. The mass was located on the radial side of the flexor tendon (arrow). (B) Following excision of the mass, preserving the neurovascular bundle (arrow). (C) The mass was excised gradually. Some parts of the mass were hard.

**Figure 5:**
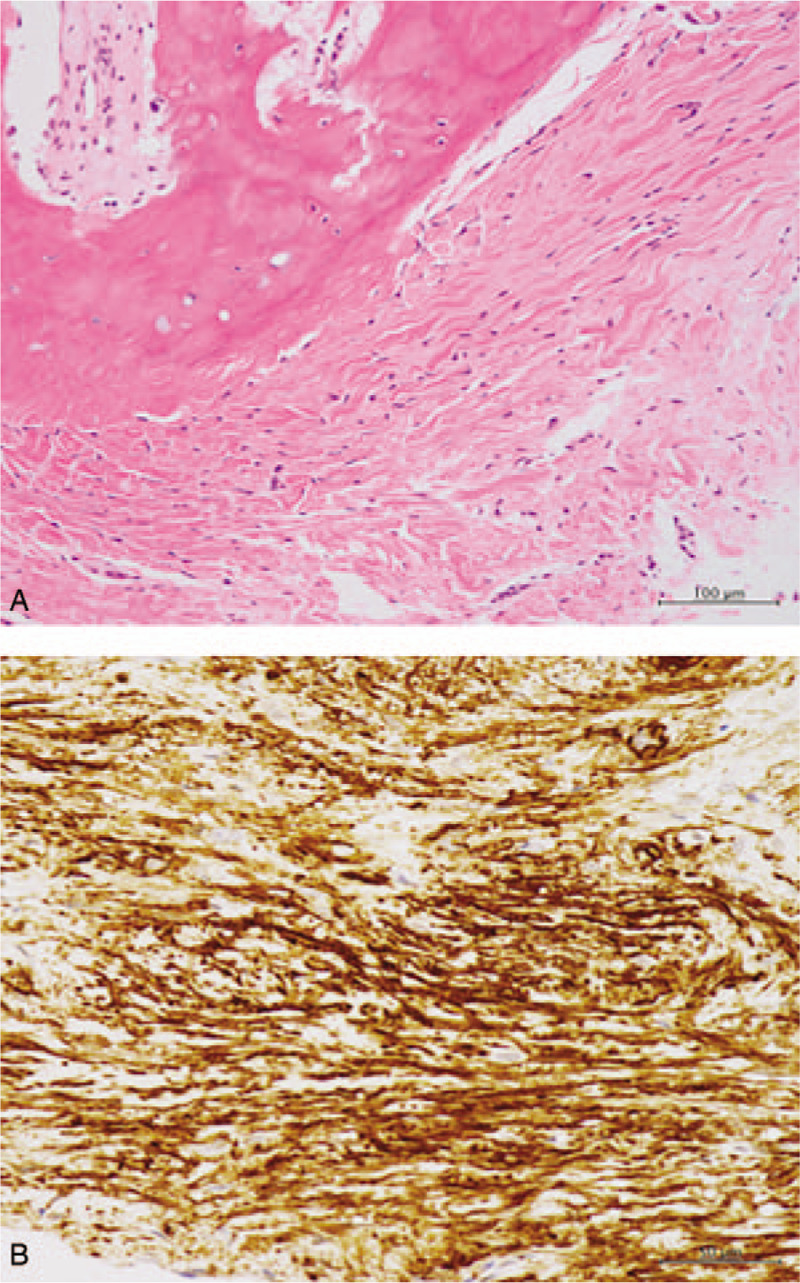
Histological findings of the resected mass. (A) Fibroblastic proliferation and scattered foci of osteoid formation. There is no atypical fission image. Hematoxylin and eosin staining, original magnification × 200. (B) The brown-stained area indicates positivity for ASMA. ASMA immunostaining, original magnification × 100. ASMA = alpha-smooth muscle actin.

**Figure 6:**
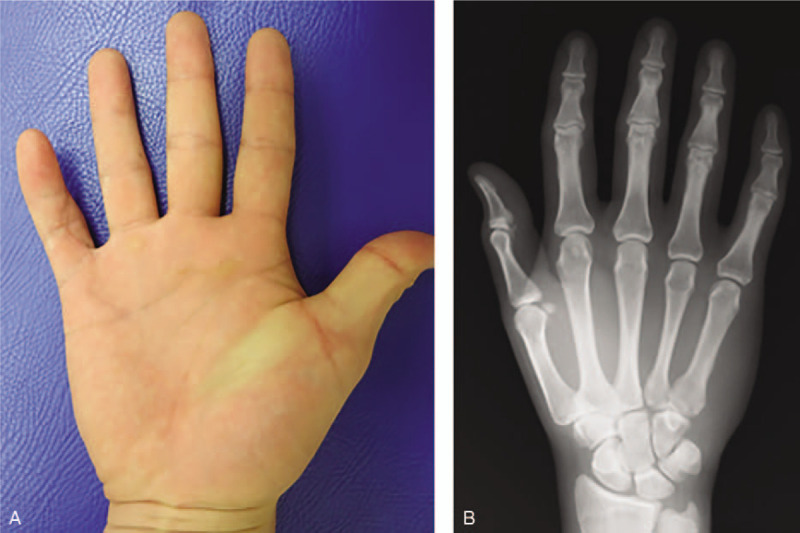
The hand of the patient 4 weeks after the surgery. (A) There were no wound complications following surgery. Radiological investigation at postoperative 6 months (B) did not show swelling or calcification.

The patient underwent surgery for exploration and excision of the mass. The possible risks associated with this intervention were explained to the patient. Exploration revealed a subcutaneous fibrous mass with hard components in some places that was attached to the flexor tendon and the tendon sheath (A2 pully) (Fig. 4A). As revealed in the preoperative assessment, the mass was adjacent to the radial neurovascular bundle. The mass was gradually excised, preserving the radial neurovascular bundle (Fig. 4B and C). The A2 pully was attached to the mass; hence, it was also resected. Histological assessment showed a lesion with fasciitis-like features, fibroblastic proliferation, and scattered foci of osteoid formation that was positive for alpha-smooth muscle actin (ASMA) 1A4 immunostaining, without evidence of malignancy (Fig. 5A and B). Postoperatively, the wound healed without complications (Fig. 6A). At postoperative 6 months, there were no signs or symptoms of recurrence (Fig. 6B), and the patient had returned to his premorbid functional status.

## 3 Discussion

FOPD is a rare benign lesion that occurs in the subcutaneous tissue of the digits. Histopathologically, it is characterized by fibroblastic proliferation with foci of osseous differentiation,^[1,3,4]^ and predominantly affects young adult females.^[4]^ The etiology of FOPD remains unclear. Although a history of trauma was not related to diagnosis in this case, such history may occasionally be reported by patients with FOPD.^[1,4,5]^ In the studies conducted by Dupree and Enzinger,^[1]^ a history of trauma was associated with the lesion in only 9 of 21 examined patients; moreover, both a neglected minor injury and infectious process have to be considered as possible causes. In the present case, there was no history of associated trauma or infection. However, 4 weeks earlier, our patient underwent resection of a mass in the same region. Therefore, we cannot rule out the possibility that the trauma caused during the previous surgery may have played a role in the development of the present lesion. Alternatively, if the previous lesion was partly excised at an early phase of its growth, it is possible that our patient developed local recurrence. Finally, the occupation of this patient involves extensive use of the little finger; thus, it is reasonable to speculate that this factor may have been involved in the development of the mass.

The most important differential diagnosis in this case was extraskeletal osteosarcoma; the rapid growth led to a strong clinical suspicion of a malignant tumor. One of the main defining features, yet not specific, was the age of the patient at presentation. FOPD usually affects young adults aged 20–30 years; conversely, extraskeletal osteosarcoma is rarely observed in patients aged <35 years.^[6]^ Moreover, extraskeletal osteosarcoma rarely affects the digits. Histologically, it is characterized by neoplastic osteoid and nuclear atypia.^[4,5]^

Other major differential diagnoses include myositis ossificans, bizarre parosteal osteochondromatous proliferation (BPOP), and subungal exostosis. The clinical and histological features of myositis ossificans are similar to that of FPOD. Some investigators have suggested that FOPD and myositis ossificans are in the same disease spectrum, but they occur in different locations.^[5,7,8]^ Myositis ossificans is more commonly observed in young males and often occur after trauma^[4]^; moreover, it is typically found deeper in the soft tissue.^[9]^ Furthermore, myositis ossificans is histopathologically characterized by zonation pattern with a fibroblastic tissue core or base and a superficial rim of osseous tissue. However, magnetic resonance imaging assessments are typically better in showing zonation patterns prior to the appearance of ossification.^[10]^ BPOP, similar to FOPD, arises more proximally in the digits of the hand than in the feet.^[11]^ However, on radiological assessment it is observed that BPOP attaches to the bone through a stalk and via pathologic demonstration of a cartilaginous cap. FOPD is considered as an extra-osseous lesion and typically lacks a cartilage intermediate. Subungal exostosis is clinically and histopathologically similar to FOPD, except for the presence of connection to the underlying bone (radiological assessment) and the presence of a cartilaginous cap (histological assessment).^[5,9]^

In our case, radiological investigation revealed that the lesion presented as an ill-defined soft tissue mass with focal calcification, lacking the typical geographic distribution or zoning patterns of myositis ossificans. The surrounding calcifications were not continuous with the proximal phalanx bone. Histologically, the lesion consisted of a mixture of fibroblasts, osteoblasts, osteoid formation and calcification. Fibroblast transition to osteoblasts and osteocytes, as well as the transformation of collagen to osteoid and bone, are also common features observed under the microscope. However, osteoblasts do not show cellular atypia, and the presence of osteoclasts and bone marrow elements is rarely noted. Immunostaining analysis of such lesions typically yields focal positivity for ASMA, S100, and CD34 markers.^[5]^ In this patient, histological assessment of the excised mass revealed similar findings, including scattered osteoid formation, myofibroblast proliferation, and positivity for ASMA, S100, and CD34 immunostaining.

Currently, complete excision of the lesion is the standard treatment choice for treating FOPD. After complete excision, this lesion is linked to favorable prognosis, with a low risk of local recurrence and no reports of malignant transformation.^[1,3]^ However, incomplete excision or malignant findings related to the lesion have been associated with a higher chance of recurrence.^[1,3,9]^ In our patient, we considered the possibility that incomplete excision during previous surgery at another hospital may have led to recurrence or that the rapidly growing tumor had undergone malignant transformation. Therefore, patient follow-ups are recommended in such cases. Any evidence of tumor recurrence usually indicates the requirement for surgical re-excision and, in some cases, ray amputation of the finger.^[1,9]^ In our patient, preoperative radiological imaging was immensely helpful in identifying the nature of the mass extension and involvement of neurovascular bundles. Meticulous dissection and handling assisted in optimizing the surgical outcome without residual effects on any vital structures.

In conclusion, it is important for clinicians and pathologists to recognize the presence of FOPD and treat such lesions accordingly. Accurate diagnosis will avoid the use of unnecessary aggressive surgical resection.

## Author contributions

All authors were responsible for the study concepts. Data acquisition, analysis, and interpretation were undertaken by TS, TK, RS, TF, and NA. Drafting of the manuscript was the responsibility of TS, TK, RS, TF, and NA. TS, TK, RS, TF, and NA approved the final version of the manuscript to be published, and TS, TK, RS, TF, and NA have agreed to be accountable for all aspects of the work.
